# Mass elevation and lee effects markedly lift the elevational distribution of ground beetles in the Himalaya-Tibet orogen

**DOI:** 10.1371/journal.pone.0172939

**Published:** 2017-03-24

**Authors:** Joachim Schmidt, Jürgen Böhner, Roland Brandl, Lars Opgenoorth

**Affiliations:** 1 Institut für Biowissenschaften, Allgemeine und Spezielle Zoologie, Universität Rostock, Universitätsplatz 2, Rostock, Germany; 2 Department of Ecology, Philipps-Universität Marburg, Karl-von-Frisch Strasse, Marburg, Germany; 3 Institute of Geography, Universität Hamburg, Bundesstrasse, Hamburg, Germany; University of Delhi, INDIA

## Abstract

Mass elevation and lee effects markedly influence snow lines and tree lines in high mountain systems. However, their impact on other phenomena or groups of organisms has not yet been quantified. Here we quantitatively studied their influence in the Himalaya–Tibet orogen on the distribution of ground beetles as model organisms, specifically whether the ground beetle distribution increases from the outer to the inner parts of the orogen, against latitudinal effects. We also tested whether July temperature and solar radiation are predictors of the beetle’s elevational distribution ranges. Finally, we discussed the general importance of these effects for the distributional and evolutionary history of the biota of High Asia. We modelled spatially explicit estimates of variables characterizing temperature and solar radiation and correlated the variables with the respective lower elevational range of 118 species of ground beetles from 76 high-alpine locations. Both July temperature and solar radiation significantly positively correlated with the elevational ranges of high-alpine beetles. Against the latitudinal trend, the median elevation of the respective species distributions increased by 800 m from the Himalayan south face north to the Transhimalaya. Our results indicate that an increase in seasonal temperature due to mass elevation and lee effects substantially impact the regional distribution patterns of alpine ground beetles of the Himalaya–Tibet orogen and are likely to affect also other soil biota there and in mountain ranges worldwide. Since these effects must have changed during orogenesis, their potential impact must be considered when biogeographic scenarios based on geological models are derived. As this has not been the practice, we believe that large biases likely exist in many paleoecological and evolutionary studies dealing with the biota from the Himalaya-Tibet orogen and mountain ranges worldwide.

## Introduction

One of the most basic and general biogeographic patterns is the elevational increase in species ranges, species assemblages, and ecosystems from the poles to the equator [1], largely driven by the latitudinal temperature gradient. The importance of temperature compared to other abiotic factors, e.g., precipitation and geology, varies among species, assemblages, and ecosystems but is naturally highest for temperature-determined patterns, such as tree lines [2,3]. The tree line, which per definition represents the lower border of the alpine belt, occurs at surprisingly uniform growing-season temperatures worldwide [4]. It is thought that the main reason for the position of the tree line is the heat deficit in the soil and above-ground air [4]. Other types of biogeographic drivers, e.g., annual precipitation and thermal sums, do not explain the location of tree lines on a global scale [4,5].

Nevertheless, this pole-to-equator gradient of tree and snow lines with increasing elevations is modified by a combination of the mass elevation effect (MEE) and the lee effect (LEE) [6]. Because of these effects, elevational belts are generally located higher in the inner parts of mountain systems than along the edges. MEE was first introduced by Quervain [6], who described the observation that tree lines occur at higher elevations in the central European Alps than at their fringes. This concept has been extended to all temperature-based parameters (e.g., snow lines) and generally to all mountain ranges. Physically, it is based on the higher average air temperature above the ground of highlands than at the same level in the free atmosphere above lowlands due to the heating effects of the surfaces. This effect has been comprehensively described both climatologically and geomorphologically [7]. LEE in turn describes the mountain massif’s shielding of leeward parts from clouds and precipitation, with increased temperature owing to Föhn effects and higher solar radiation. Thus, the strength of the influence of these two effects on elevational patterns should increase with the importance of temperature and/or clouding for the biogeographic distribution.

Although these two climatological effects have been described long ago, to our knowledge no studies have quantified their influence on organisms other than trees [8] or on phenomena other than snow lines [9]. Thus, the generality and extent of the biogeographic impact of these two effects is not yet known. This is easily documented by an ISI search with the term ‘mass elevation effect’ that produces only seven hits, all of which deal with tree and snow lines. This is in itself a significant gap in our understanding of large-scale biogeographic patterns and processes. In addition, we believe that this knowledge gap often leads to considerable bias when paleoecological and evolutionary patterns are interpreted because the size, dimension, and latitudinal position of orogens, and thus MEE and LEE, change on evolutionary time scales. Consequently, ignoring MEE and LEE will result in wrong conclusions regarding paleodistributions of species and biomes, faulty conclusions regarding past climatic conditions, and thus wrong predictions of the consequences of future climate change.

In this study, we utilized distribution data on ground beetles in the Himalaya-Tibet orogen (HTO) to analyze the impact of MEE and LEE on the distribution ranges. There, MEE should be especially pronounced since the HTO is the largest mass elevation on Earth and is situated in subtropical latitudes, and thus receives high insolation throughout the year [10]. LEE is always combined with MEE and significantly increases its influences on high-altitude ecosystems [10]. The high-elevated mountain arc of the Greater Himalaya along the southern margin of the Tibetan Plateau acts as an effective barrier to the northward-streaming Indian summer monsoon [11] and thus enormously reinforces LEE in the orogen. Along the southern margin of the orogen, LEE is assumed to be climatically more important than MEE [12]. The quantitative contributions of both effects to the local radiation and thermal climate of the region are, however, difficult to estimate precisely [6]. Ignoring LEE, Han and colleagues [8,9] investigated the quantitative contributions of latitude, longitude, and MEE to the elevational position of the snow line and timberline in both Central and East Asia. The authors found that latitude and mountain base elevation (representing MEE to some extent) are important factors controlling the snow line. According to their study, the mountain base elevation explains ~57% of the variance of the snow lines south of latitude 32°N (south of Central Tibet). Likewise, latitude and mountain base explain ~46% on average of the variance of timberline elevation in Central and East Asia [9]. It is therefore not surprising that the elevation of vegetation zones markedly increases against the latitudinal trend along an imaginary south–north transect from the Himalaya to South Tibet. For example, the tree line rises from 3,600–3,900 m a.s.l. on the southern slope of the eastern and central Himalaya (~27.5–28°N) to about 4,600–4,800 m in the Transhimalaya (~30°N [5,13,14]).

Ground beetles (Coleoptera: Carabidae) generally have temperature-driven faunal distributions [15–17]. The species used in this study are additionally characterized by a markedly restricted dispersal ability, as indicated by primary winglessness (i.e., winglessness by descent). As a consequence, they remain at their high-altitude habitat at all stages of their life cycle and should be thus an ideal proxy to test the impact of MEE and LEE on the elevational ranges of soil-living arthropods in general. They are also ideally suited for this purpose because knowledge of their taxonomy, economy, and distribution is the most advanced among the epigaeic fauna in the HTO (e.g., [18–25].

In this study, we tested whether the elevational distribution of high-alpine epigaeic fauna, specifically ground beetles, shows similar patterns and is triggered by the same climatic effects as tree lines and thus increases following MEE and LEE from the south face of the Greater Himalaya to the Transhimalaya. In addition, we tested whether July temperature as a proxy for seasonal temperature and solar radiation as a proxy for soil temperature are good predictors of the respective elevational ranges of the alpine ground beetle species of the orogen. Finally, as MEE and LEE have changed during orogenesis, we provide a general assessment of the importance of these effects for describing the distributional and evolutionary history of the orogen biota.

## Methods

### Study area

**Fig 1:**
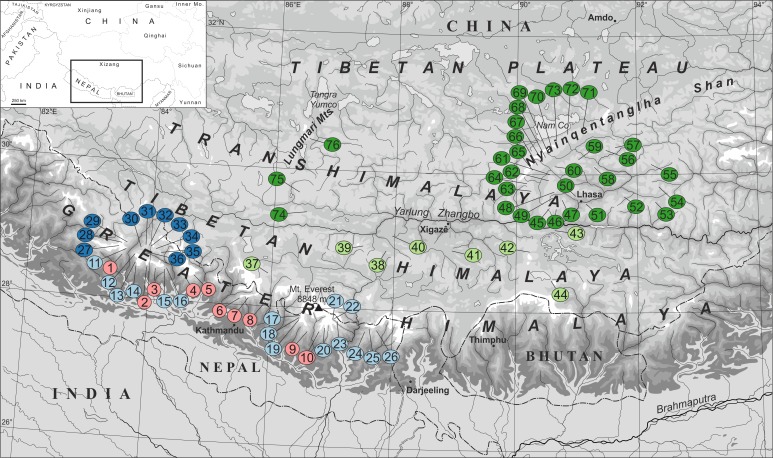
Map of the southern central Himalaya–Tibet orogen showing the main features of the mountain system and the positions of the 77 investigated locations of high-alpine ground beetle fauna.

The study area (Fig 1) encompasses the southern central portion of the HTO where the influences of MEE and LEE on the tree line, timberline and snow line are highest [5,8,9]. Furthermore, this is the portion of the HTO where the distribution of ground beetles has been most intensively investigated (e.g., [18–25].

### Climate data

Spatially highly resolved climatic measurements are not readily available for most if not all mountain massifs in the world. As a consequence, we used modelled temperature data (see below). Beetle distributions are strongly linked to soil temperature [15,16], but even less information on this variable is available than information on air temperature. Solar radiation is one of the most important determinants of soil temperature, flanked by convection, conduction, and evaporation [26]. We therefore used solar radiation as a surrogate for soil temperature and thus as an additional proxy for the elevational ranges of the beetle species. Furthermore, as solar radiation is directly influenced by cloud cover, it likely reflects LEE better than air temperature does.

Spatially explicit estimates of monthly mean temperatures were modelled on a regular grid network with a grid-cell spacing of 1 km^2^ using Model Output Statistics (MOS) downscaling of ERA-Interim reanalyses [26–28]. ERA-Interim temperatures with 0.7° (lat/long) resolution were spatially refined according to the altitude-adjustment and bias-correction procedure proposed by Gerlitz et al. [27]. Biases in the temperature fields were quantified and corrected taking available time series of observed daily mean temperatures of 78 weather stations into account [27]. The suitability, precision, and limitation of the downscaling approach for the Tibetan Plateau and the Himalaya is discussed in Gerlitz [28] and Gerlitz et al. [27,29].

Because homogenous radiation records from the HTO are widely lacking, solar radiation values were taken from Böhner [26]. The gridded 1 km^2^ resolution data set of monthly shortwave topographic irradiation (i.e., diffuse and direct shortwave solar radiation on inclined surfaces) was modelled using a semi-empirical approach. In the first step, clear sky atmospheric extinction of solar radiation was estimated by integrating the Bouguer-Lamberts equation within a simple atmosphere mass parameterization scheme, and cloud-induced reduction of radiation was subsequently calculated using the Ångström equation [26]. To identify diurnal phases when the horizon shelters the surface from direct irradiation, topographic horizons were computed as the maximum elevation angle in the direction of the sun’s azimuth angle. The entire procedure was performed with an integration frequency of 60 min and considered sun-ray refraction (for details, see [26,30].

### Ground beetles as a model group

We used extant ground beetles (Coleoptera: Carabidae) as a model group to assess the impact of MEE and LEE on their respective elevational ranges. The ground beetle species considered in this study are primary wingless (winglessness by descent, indicated by shortened metathoracic plates) and have therefore a markedly restricted dispersal ability. Populations of the wingless ground beetle taxa do not change their respective distribution during seasons or at life stages. Thus, the respective elevational ranges recorded at any given time during the year reflect the respective extant elevational range limits of that species.

It has been proposed that temperature is one of the key climatic factors determining beetle distribution [15,16,31]. Thus, the zonal distribution is determined by the respective lower and upper temperature limits of each species during its main activity period (average summer temperatures in latitudes with seasonal climate). Accordingly, in mountain regions, the limits of the respective elevational ranges of beetles are determined by species-specific temperature limits.

We used elevational range data of ground beetle species from the Himalaya-Tibet orogen. As we aimed at comparing our results with published results on the tree line, and to ensure that our approach is tree-line independent, we only used the distribution data of high-alpine species (as opposed to low alpine species, which per definition are linked to the tree line). Elevational ranges are defined by their upper and lower range limits. We only used the lower limit of the species-specific elevational ranges. The rationale for focusing on the respective lower range limits is that the upper range limits often are blurred by local geographic conditions, for example when bedrock is covered by thick layers of scree unsuitable as a ground beetle habitat or when upper range limits reach the local mountain crest. For the selection of high-alpine species, we used two different strategies. In the first, a species was classified as high-alpine when its elevational range is centered within the high-alpine zone as defined by Miehe [13] for the Himalaya and South Tibet and when its lowest occurrence is situated distinctly (at least 100 m) above the lower border of the lower alpine zone [13]. In the second strategy, we used the 15 species ranges of each geographic unit with the highest lower range limits. Both data sets were analyzed identically.

### Assessment of distribution data

The respective distribution patterns of ground beetles in the Himalayan–Tibetan orogen (S1 Table) have been investigated by one of the authors (J.S.) since 1992 in the context of faunistic, taxonomic, and phylogenetic studies. For this current macroecological study, no new fieldwork was conducted. Instead, the distribution data from the Schmidt High Asia Carabidae Data Base (SCDB) were used. Nevertheless, to show that the available distribution data are adequate for the current macroecological study, we describe in detail how the original fieldwork had been conducted.

Fieldwork for the SCDB took place during the period of main activity of the imagines from May to September in 27 biogeographic surveys in the Nepal Himalaya and in 5 surveys in Tibet between 1992 and 2015. To confirm the sampling results, some of the sites were investigated twice in different years or different periods of a year (beginning and end of the imagines’ season). Unlike in ecological studies, where the need for abundance data require quantification by means of standardized ecological methods, the SCDB is a faunistic database based on complete qualitative assessments of the ground beetle species of the high-alpine zone. This was possible because of the knowledge of the particular habitat preferences of the respective species. During fieldwork, epigaeic alpine species were systematically searched for under stones and in rock debris along slopes with appropriate soil conditions. For each of the investigated high mountain areas, data were collected for the complete diversity of alpine habitats suitable for ground beetles, specifically humid slopes, meltwater channels, gullies, and older moraine sites. This method allows a direct comparison of the respective sampling sites and was successfully applied to the alpine ground beetle fauna of southern Tibet by Schmidt et al. [32] in the investigation of range shift driven by climate change since the last glacial maximum.

Since ground beetles are able to survive in the high-alpine environment only with sufficient soil humidity, occurrences of these beetles are restricted to very few places along the mountain slopes and high valleys, specifically margins of meltwater channels and puddles, groundwater withdrawals, late melting snowfields, and knolls of older moraines. Consequently, these appropriate sites were not subsampled but were instead assessed completely. At such sites, the species usually achieve high abundances and are easy to detect under stones, where individuals often accumulate during daytime. The activity of high-alpine beetles on the soil surface is, however, very low (*Amara*, *Bembidion*, *Nebria*) or absent (strictly edaphic *Deltomerodes* and *Trechus*). In addition, the high-alpine zone is influenced by heavy snowfalls also during the summer. Therefore, standard techniques of ground beetle ecology (e.g., pitfall trapping) would result in insufficient results; hand collecting is the appropriate sampling method. This method allows a nearly complete assessment of the species composition of the alpine ground beetle fauna of the respective slopes, and errors in determining the species-specific elevational limits of distribution are considered to be low (< 50 m difference in altitude). This assumption is supported by the fact that the same species and distribution patterns were found when sampling was repeated in different years. The respective elevational limits of distribution along the different mountain slopes were documented using a barometric altimeter and GPS. Distribution data of 118 species from 76 high-alpine locations were collected. In addition, distribution data of ground beetle species collected by other researchers on the same mountain slopes were also considered when their respective location data were sufficiently precise, i.e., when locations were exact or when GPS data had a possible error in altitude less than 50 m. All specimens were determined to the species level by J.S. using the most recent species group revisions (see supplementary S1 Table for details). Publication of the formal names of the new species (in S1 Table indicated by “sp.n.”) is part of an ongoing systematic review of the relevant species groups by the working group of J.S. and will be published elsewhere. Until the species names are published, information on these species can be directly requested from J.S.

The distribution data were grouped according to the geographic positions of the studied locations in the orogen along an approximate south–north transect according to the assumed increasing influence of MEE and LEE (Fig 1): south slope of the Greater Himalaya, main ridge of the Greater Himalaya, north face of the Greater Himalaya, Tibetan Himalaya, and Transhimalaya (10, 16, 10, 8, and 33 locations, respectively).

### Data exploration and statistical analyses

All statistical analyses were carried out using the R system for statistical computing, version 3.1.2 [33] with the packages lme4 [34], and AID [35]. Data exploration was done by visual inspection of outliers, homogeneity, and normality of residuals by means of Cleveland dotplots, conditional boxplots, and QQ-plots, respectively, as well as by using the boxcoxnc function of the AID package. Where necessary, data were box-cox transformed.

### Correlation of distribution ranges with temperature

We correlated average July temperature as fixed effect with the lower elevational distribution limit as response variable and used species as random effect to reflect the recording of some species at multiple sites by fitting a linear mixed effects model to the data. Theory predicts that MEE and LEE should lead to an increase in temperature and lower distribution limits towards the interior of the mountain massif. We therefore included an additive term of temperature and location in the model. At the same time, LEE should lead to an abrupt decrease in the slope of this effect in the transition from the Northern Himalaya to the Tibetan Himalaya as the impact of changes in cloud cover abruptly ends. We therefore also included an interaction term between temperature and location (mountain ranges). Both terms were included as fixed effects. This model was then simplified using Aikaike information criteria (AIC).

### Correlation of distribution ranges with solar radiation

As solar radiation should already reflect MEE and LEE, a simple linear function should adequately reflect the correlation between solar radiation and the elevation of the lower elevational distribution limits. Thus, we only fitted a simple linear mixed effects model to this data, incorporating solar radiation as fixed effect and species as random effect. Marginal R^2^ (proportion of variance explained by the fixed factors alone) and conditional R^2^ (proportion of variance explained by both the fixed and random factors) were calculated following Nakagawa and Schielzeth [36,37] and Johnson, as implemented in the R script of Lefcheck (http://jonlefcheck.net/2013/03/13/r2-for-linear-mixed-effects-models/).

### Correlation of distribution ranges with mountain interiority

By definition, MEE should increase with mountain interiority. As the orogen is oriented roughly east–west, the latitudinal position can function as an approximation of mountain interiority. Thus, we used the latitude of each sampling site as a proxy for mountain interiority and plotted it against elevation and solar radiation. Again, we predicted that solar radiation, and thus temperature and altitudinal distribution of the beetles, should strongly rise in the beginning when MEE and LEE are superimposed, and then their slopes should decrease once cloud cover has no effect. In this model, distance was treated as fixed effect and species were again treated as random effect.

### Comparison of distribution ranges among orogens

In the final step, we tested whether elevations in the five investigated geographic sections of the Himalayan south slope to the inner portions of the orogen vary significantly by means of an ANOVA using species as the error term to reflect the recording of some species at multiple sites.

## Results

### Data on species distribution ranges

The distribution range data are homoscedastic, and no outliers were detected. The residuals were normally distributed. However, as expected, the combined distribution range data were left-skewed. Consequently, we ran all linear models with and without a box-cox power transformation. Since the results were practically identical, we only report the models with the non-transformed parameters.

### Correlation of distribution ranges with temperature

**Fig 2:**
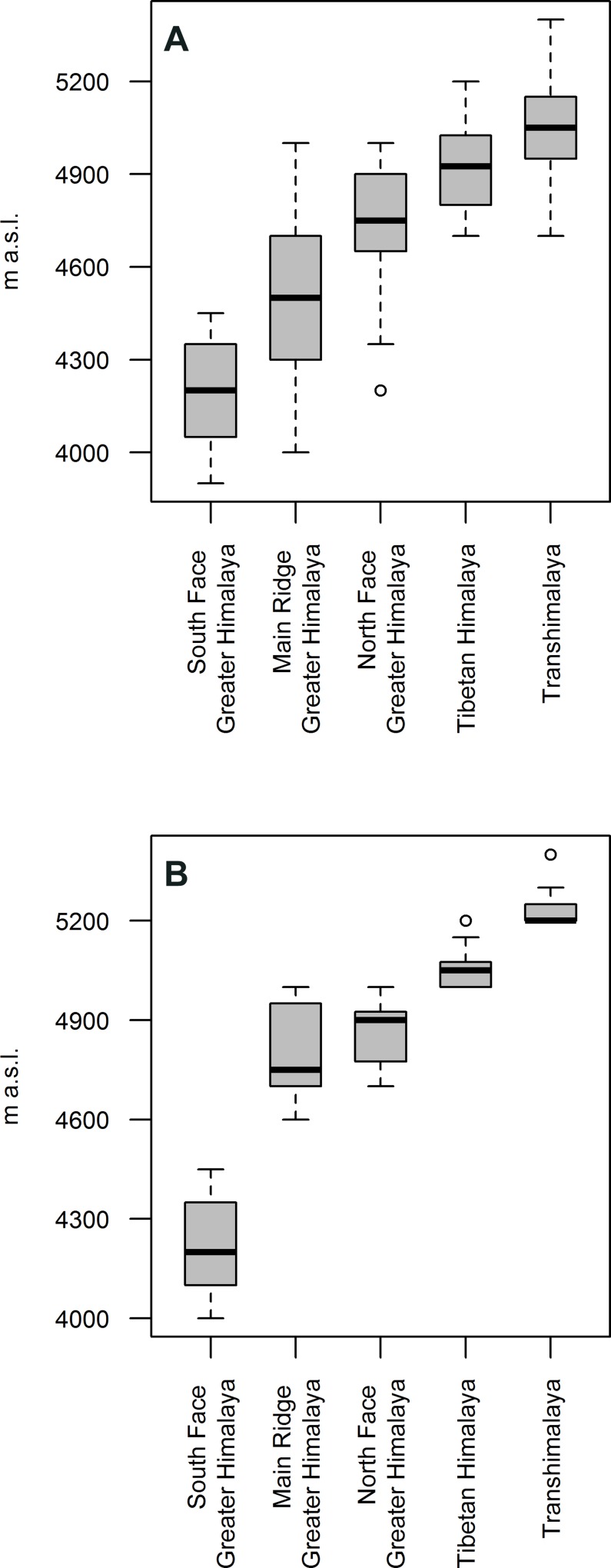
Boxplots of the lower border of the elevational distributions of (A) all 118 records of high-alpine ground beetle distribution ranges in 5 different parts of the southern central Himalaya–Tibet orogen, sorted geographically from south (left) to north (right); and (B) the highest 15 ranges of all 5 mountain ranges.

**Fig 3:**
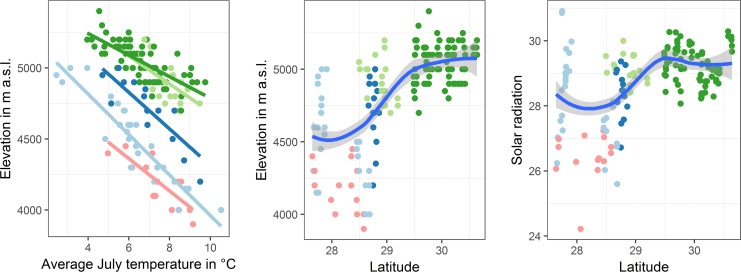
Lower range limits of elevational ranges of alpine ground beetle species across the main mountain ranges of the southern central Himalaya–Tibet orogen plotted as a function of (A) the average July temperature and elevation, (B) latitude and elevation, and (C) latitude and solar radiation.

According to the lowest AIC value, the model that best fit the data included an interaction term between temperature and location and included species as random effect (S2 Table). As expected, this linear mixed effects model revealed a significant positive effect of July temperature and location on the respective lower limits of the distribution ranges of alpine beetles (marginal R^2^ = 0.90, conditional R^2^ = 0.94); the respective species elevation ranges of the beetles increased with increasing temperature and from the south slope of the Greater Himalaya to the Transhimalaya (Fig 2A). Furthermore, even though the elevations of the lower limits of distribution varied markedly from species to species within the respective parts of the orogen (Fig 2), the elevations of ranges in five investigated geographic sections from the Himalayan south slope to the inner portions of the orogen varied significantly (F_4, 118_ = 85.4, p < 0.0001, Figs 2A and 3A). The same held true when only lower elevational limits of the 15 highest ground beetle species for each of the five geographic units were sampled (F_4, 45_ = 119, p < 0.0001, Figs 2B and 3B), although this latter result needs to be considered with caution given that the data does not meet the assumption of homogeneity of the variances.

The interaction term in the linear mixed effects model revealed a significant interaction effect of July temperature and location at the lower limits of the distribution ranges. Accordingly, the shift in the lower limits of distribution from the southern to the central orogen is not linear (Fig 2B).

**Fig 4:**
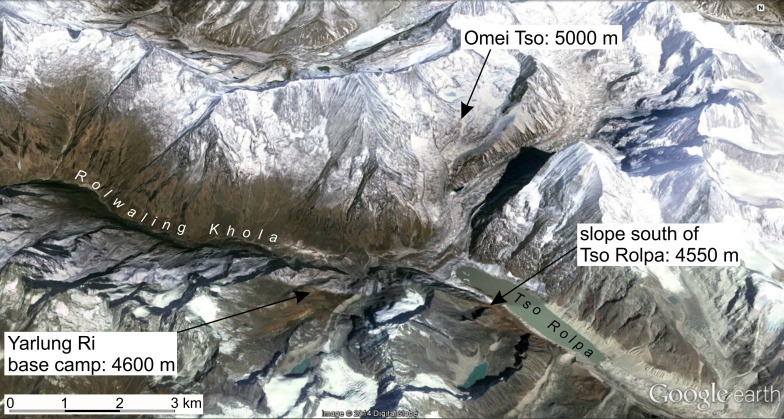
Relief map of the upper Rolwaling Valley in the Greater Himalaya of eastern Central Nepal showing the geographic position of three separated sites with populations of high-alpine ground beetles (for additional information, see Fig 1 and S1 Table, location numbers 17–19). The elevation data added to each of the locations correspond to the lower limit of the elevational distribution of the species *Nebria molendai*, which was found at each of these locations. On the warmer south-exposed slopes, representatives of the high-alpine ground beetle fauna seemed not to occur below 5,000 m a.s.l., while on the slopes, the species can be found 400 m lower. The north-exposed slopes are shadowed by surrounding mountains until late morning and later on by the usually early arising clouds around the mountain tops. This situation results in colder average soil temperatures during summer and a shorter season owing to the much later snowmelt on the north- and west-exposed slopes.

According to AIC values, the inclusion of species as random effect again increased the fit of the model significantly (S2 Table). As expected, the linear regression model revealed a direct significant positive correlation of solar radiation with the elevation of the distribution ranges (marginal R^2^ = 0.59, conditional R^2^ = 0.79, Fig 3). As expected from cloud cover regimes, also solar radiation steeply increased in the initial succession of the mountain ranges and then fell off towards the Tibetan Himalaya and Transhimalaya (Fig 4C).

## Discussion

### Ground beetle distribution ranges

Our data indicated that MEE in combination with LEE causes the high-alpine ground beetle fauna to raise their elevational distribution limit against the latitudinal gradient from the southern margin of the HTO 200–250 km northwards to the interior of Tibet, with a pattern resembling that of tree lines and snow lines in the orogen. For example, the median elevation of the species distribution ranges on the Himalayan south face lies 800 m lower than that of the Transhimalaya (Fig 2). Because of this enormous difference, the elevational areas of the high-alpine ground beetle faunas of the inner and southernmost portions of the orogen do not overlap. In the Transhimalaya, the lowest occurrences of high-alpine species were usually found above 4,800 m a.s.l. On the other hand, this altitude on the southern slope of the Greater Himalaya is characterized by exposed bedrock, scree, firn, or glacier, and thus clearly lies in the nival zone. On this slope, the highest occurrences of ground beetles were never above 4,600 m a.s.l. By contrast, in the Transhimalaya, ground beetles were found up to 5,600 m a.s.l., which marks the highest occurrences of beetles worldwide [30]. Overall, the increase in the median elevation of beetle occurrences (800 m) fell in a range similar to that of tree lines, i.e., to 700–1,200 m [5,13,14]. Because high-alpine ground beetles are independent of the tree line, the congruence of the data indicates that this is a general pattern valid for organisms that show little range mobility and no habitat changes in their life cycle and whose habitats are strongly determined by the temperature regime close to the ground.

### MEE and LEE

The presented biogeographic data from the southern margin of the HTO are of particular interest for discussing the regional differences in MEE and LEE on high-altitude ecosystems. The medians from the south face to the north face of the Greater Himalaya steeply increased; at a distance of only 20–30 km, the medians already differed by ~500 m, which is almost two-thirds of the difference across the entire investigated area, and reflects the sudden decrease in LEE in Tibet. Across the remaining 200 km of the south–north transect, the limits of distribution of the high-alpine fauna thus increase considerably slower from the north slope of the Greater Himalaya towards the Transhimalaya. Thus, our ground beetle data and tree-line data [5] suggest that about 60% and 40–50%, respectively, of the total increase in the average summer soil temperature occurs within the narrow mountain chain of the Greater Himalaya, which covers only 10% of the entire latitudinal transect. This fits nicely with observations of Miehe [14], who noted a 500 m ascent of the borderline between the lower and higher alpine vegetation zone from south to north across the Himalayan main chain. We found the same value for ground beetles in this area.

**Fig 5:**
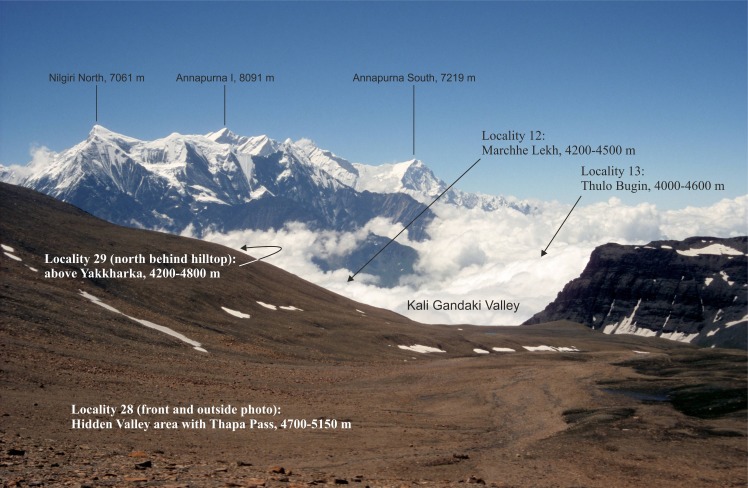
View from Thapa Pass (5,250 m a.s.l.) towards the southeast across the Kali Gandaki Valley, with the Annapurna mountain range in the background (central Nepal Himalaya, 11 July 1998 at 10:00 a.m.). Northwards-streaming humid air masses of the Indian summer monsoon are dammed on the south face of the Greater Himalaya but penetrate far into the inner portions of the orogen along the Himalayan transverse valley. While the alpine zone on the Himalayan southern slopes and on the slopes along the Kali Gandaki Valley are densely covered by clouds almost continuously during monsoon season, the Thapa pass and adjacent Hidden Valley (outside the photo) receive high solar radiation until late morning almost every day because this part of the orogen lies in the rain shadow of Dhaulagiri I (8,167 m a.s.l.). These differences in local climate result in distinct differences in the elevational patterns of distribution of high-alpine ground beetles at very short distances. Four examples are indicated (locations 12, 13, 28, 29), along with the lower and upper limits of distribution of the respective species composition.

When we considered the 15 highest species distributions, this pattern was even more pronounced as it prunes the lower species from the very large elevational spread of distributions along the main ridge of the Greater Himalaya (up to 1,000 m). In this portion of the orogen, the extremes include almost the entire range of values found on the south and north faces of the Himalaya together. By contrast, when we compared the distribution data from the south and north faces of the Greater Himalaya, the range of overlapping values was very small. Along the main ridge, remarkable differences in the positions of the lower limit of distribution were found even between locations that are very close to each other but on opposite slopes, e.g., in the Rolwaling Valley of the eastern Central Nepal Himalaya (Fig 5). As could be expected, slope exposure, and with that solar radiation and ground temperature, seemed to be some of the main reasons for the spreading of the values in all parts of the orogen.

**Fig 6:**
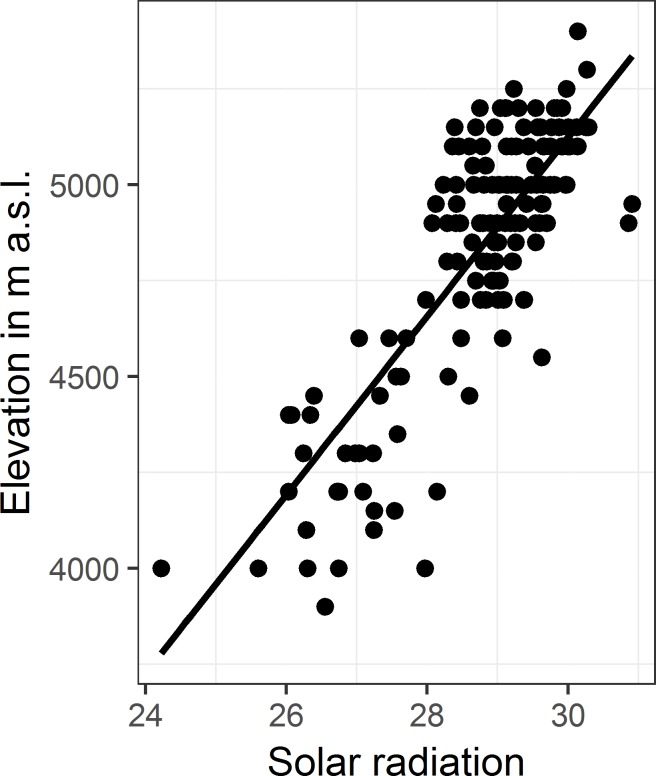
Lower range limits of elevational distributions of alpine ground beetle species across the main mountain ranges of the southern-central Himalaya–Tibet orogen plotted as a function of solar radiation.

Remarkable differences in the elevational positions of ground beetle distributions were also found between adjacent locations when cloud conditions of these sites differed consistently. Along the Kali Gandaki Valley, which is a Himalayan transverse valley between the stretching massifs of Dhaulagiri and Annapurna above 8,000 m a.s.l., monsoonal clouds can reach deeply into the inner portions of the Himalaya (Fig 6). Thus, during summer, the mountain slopes on both sides of the valley are almost continuously covered by heavy rain clouds. By contrast, on the leeside of the bordering mountains, the sky is clear on most days, at least during morning, with respective effects on solar radiation and thus ground temperature on both sides. According to these climatic differences along the mountain slopes within the Kali Gandaki Valley, the lower limits of distribution of high-alpine Carabidae are situated not higher than 4,600 m a.s.l. (locations 11–14, 29–31, Fig 1, S1 Table), while on slopes in the rain shadow of the immediately surrounding mountains (locations 27, 28, 32–36), this border lies at least 100 m higher.

Flohn [12] concluded early on, based on meteorological investigations, that LEE is the dominant effect on the southern margin of the orogen. This is due to the enormous differences in cloud cover and precipitation patterns between the southern and northern slopes of the Greater Himalaya [13][32]. These climatological processes translate directly to the biogeographic observations made along the slopes of the Himalayan transverse valleys, where monsoonal clouds occur, similar to those of the southern slopes of the Himalaya. On the southern slopes, the limits of distribution of the high-alpine species were situated generally lower than on immediately adjacent opposite slopes, which are in the rain shadow even when located more southerly (Fig 6). Such observations can only be explained by a locally stronger influence of LEE on the high-altitude ecosystems.

In contrast to the particular climatic conditions on the southern slopes of the Himalaya and along the transverse valleys, the precipitation patterns of the investigated mountain ranges north of the Himalayan main chain, including the Tibetan Himalaya and Transhimalaya, are much less different [14,38], and consequently, also the differences in the radiation patterns should be comparatively low. This leads to a much slower ascent of the limits of distribution of both the high-alpine ground beetle fauna and the tree line [5] across this part of the transect compared to that observed on the Himalayan main chain. Thus, this pattern can be explained by a gradual increase in MEE alone.

Within single parts of the orogen, the position of the lower limit of distribution of the high-alpine ground beetles varies by no more than 500–700 m (Fig 4A) except for a few extreme values. It is likely that this variation is mainly caused by the effect of slope exposure. Other factors might also expand the range of the local elevational distribution of high-alpine ground beetles, particularly along the lower limit, e.g., the cooling transport of meltwater streams or the “cold store effect” of canyons and avalanche corridors, which become free of snow late in the season.

### Air temperature and solar radiation as proxy

Even though soil temperature is the driving force for distribution ranges of ground beetles, the limited availability of soil temperature data for the world’s mountain ranges still prohibits its usage. Available temperature data are usually air temperature. Our correlation analyses of beetle elevational ranges with July temperature and location showed clear patterns. July temperature alone explained only a small fraction of the variance in distribution (data not shown). This was expected because towards the interior of the orogen, due to MEE, similar temperatures occur as elevations increase. However, when the different mountain ranges were included as interaction term, the predictive power of this combined model was very large (conditional R^2^ = 0.94). Also as expected, the interaction term was relevant due to the forcing of the abruptly weakening LEE towards the Transhimalaya.

We proposed that unlike July temperature, solar radiation should by itself be a good predictor of the elevational ranges of ground beetles without having to include location as an additional factor since it directly reflects both MEE and LEE. Our data clearly supports this notion (S3 Table). Thus, in cases where mountain interiority cannot be included as a factor, solar radiation should be superior to mere air temperature as a proxy for predicting elevational ranges across mountain ranges. This is even more noteworthy given that solar radiation is fairly easily obtained from global models. However, it would undoubtedly be even better to deal with soil temperature directly. Kearney and coworkers [39] recently published the global microclim data set, which reflects the necessity of using microclimatic data to more properly describe ecological niches for species groups. Unfortunately, these data in their current form are not suitable for mountain ranges as the resolution of 15 km^2^ per grid does not allow specification of plots in high mountain ranges. Thus, if these data were made available by, e.g., downscaling them via digital elevation models, it would be a great advancement for biogeographic studies in mountain ranges, where much of the global biodiversity is located. Until such data are available, solar radiation seems to be a very good choice for predicting temperature-driven ranges with a high spatial resolution though only in cases where the taxon’s niche mandates comparable soil moisture conditions among sites. These data will have to be flanked by ground work. For example, to assess the impact of MEE + LEE on the biota for the entire HTO, field studies are needed that take the more western (south-western Tibet) and central parts of the orogen (Changthang) into consideration. Preliminary results, however, do not indicate a further increase in the limits of beetle distribution outside the area investigated in the present study. By contrast, the highest occurrences of ground beetles worldwide are on south-exposed slopes of Nyainqentanglha Shan, central Transhimalaya (5,600 m a.s.l., at location 67 [22]), and on the same mountain with similar slope conditions, the elevation world record of Dytiscidae water beetles was ascertained (5,100 m a.s.l. [40]). Species from several other terrestrial and freshwater beetle families also occur at extremely high elevations in this area (e.g., Byrrhidae, Cantharidae, Curculionidae, Hydrophilidae, Staphylinidae, and Tenebrionidae; unpublished data of J.S.). Based on current knowledge, it can thus be assumed that as a result of the maximum impact of MEE and LEE, the Transhimalayan southern slopes provide the highest habitats for alpine arthropods worldwide.

### MEE and LEE in paleobiogeography and paleoecology

As stated, MEE and LEE have been largely neglected in biogeographic studies and even more so in paleoecological studies. Our study is ideally suited to illustrate the graveness of this negligence. If we consider that alpine environments have average July lapse rates like those areas of the orogen influenced by the Indian monsoon (0.5–0.6 K/100 m) and that the latitudinal effect on the average July temperature above land is about 5.6 K/10°N between 25 and 35°N [26], the change in summer temperature induced by the total MEE and LEE induced summer temperature effect on the alpine soil biota in the Transhimalaya is close to or slightly above 5 K. From the viewpoints of paleoecology and paleobiogeography, it is most noteworthy that this value is higher than the currently accepted temperature decline of 2–4 K of the last glacial maximum presented for the southern Tibetan Plateau [32].

In other words, the combination of the current MEE and LEE could override temperature changes of glacial cycles. Since MEE and LEE must change over the time of orogenesis in accordance with the size and position of an orogen, their impact on a given and developing biota must change as well. Consequently, especially for species adapted to colder climates, the different parts of the orogen must have been differently qualified as habitats. The potential impact of these effects therefore must be considered when different or contrasting geological models of HTO evolution [41,42] are being relied upon to derive biogeographic scenarios, and when biogeographic and phylogeographic studies are considered in discussions of geology and paleoclimatology. To emphasize the importance of these effects, one only needs to consider that today these effects alone have an impact on temperature comparable to that of an uplift of a geographically separated mountain of 800 m within the alpine belt. The importance of these effects is even more impressive if one considers that the strong Late Cenozoic global cooling [43] might have been compensated or over-compensated for. We hope that future biogeographic and phylogeographic studies will take MEE and LEE into account.

## Supporting information

S1 Table List of the observed high-alpine locations and species in the southern central Himalaya–Tibet orogen, with average July temperature and solar radiation data for the given coordinates of the respective lower limit of distribution. (DOCX)

S2 Table Comparison of random effect structures of models with temperature that either (A) included species (*N* = 118) or (B) excluded species (*N* = 232).
(DOCX)

S3 Table Comparison of random effect structures of models with radiation that either (A) included species (N = 118) or (B) excluded species (N = 232).
(DOCX)
