# Supplementary material for: Mass elevation and lee effects markedly lift the elevational distribution of ground beetles in the Himalaya-Tibet orogen
Source: PLoS One. 2017 Mar 24;12(3):e0172939. doi: 10.1371/journal.pone.0172939 (PMC5365098; doi:10.1371/journal.pone.0172939)
Supplement: S1 Table — (DOCX) [file pone.0172939.s001.docx]

Tab. 1: List of the observed high alpine localities and species in the southern central HTO, with averaged July temperature and radiation data for the given coordinates of the respective lower distributional border. Species identification is based on Hieke (2003, 2013): *Amara*; Zamotajlov (2005): *Apatrobus, Deltomerodes*; Toledano (2000, 2008): *Bembidion*; Sciaky & Wrase (1998): *Casalaeius*; Ledoux & Roux (2005), Huber & Schmidt (2009, 2012, 2013): *Nebria*; Schmidt (2009, 2016): *Trechus*.

| **locality** | **country, mountain region,** | **Carabidae species** | **species-specific** | **latitude**** | **longitude**** | **Temperature** | **Radiation** |
| --- | --- | --- | --- | --- | --- | --- | --- |
| **no.** | **locality** |  | **vertical range*** |  |  | **July [°C]** | **July [MJ/m²]** |
| **Greater Himalaya south slope** | | | | | | | |
| 1 | NEPAL, SE-slope Dhaulagiri I, | *Amara* sp.n. 1 near *jaljalensis* | 4000-(4350 m: t) | 28.580910 | 83.566148 | 8,46 | 26,74 |
|  | S-slope of Lete Pass | *Amara thorongiensis* | 3900-(4350 m: t) | 28.580444 | 83.564952 | 9,15 | 26,55 |
| 2 | NEPAL, S-slope Mardi Himal, | *Amara* sp.n. 2 near *jaljalensis* | 4000-(4450 m: r) | 28.458730 | 83.913459 | 8,11 | 26,30 |
|  | Buki Kharka | *Amara* sp.n. 1 near *franzi* | 4300-(4450 m: r) | 28.461317 | 83.916027 | 6,15 | 27,04 |
|  |  | *Trechus* sp.n. 1 near *breuningi* | 4300-(4450 m: r) | 28.461317 | 83.916027 | 6,15 | 27,04 |
| 3 | NEPAL, S-slope Annapurna II, | *Amara* sp.n. 2 near *jaljalensis* | 4100-(4200 m: ?) | 28.450298 | 84.051820 | 7,31 | 27,24 |
|  | Kiru Kharka |  |  |  |  |  |  |
| 4 | NEPAL, S-slope Himal Chuli, | *Amara manasluensis* | 4450-(4600 m: r) | 28.373041 | 84.579633 | 5,84 | 26,39 |
|  | Meme Pokhari | *Deltomerodes* sp.n. near *nepalensis* | 4450-(4600 m: r) | 28.373041 | 84.579633 | 5,84 | 26,39 |
| 5 | NEPAL, W-slope Himal Chuli, | *Amara manasluensis* | 4200-(4500 m: r) | 28.351395 | 84.739129 | 8,41 | 26,03 |
|  | Rupina La | *Deltomerodes chulii* | 4400-(4500 m: r) | 28.353426 | 84.738975 | 7,15 | 26,34 |
|  |  | *Nebria* sp.n. 1 near *orestias* | 4200-(4500 m: r) | 28.351395 | 84.739129 | 8,41 | 26,03 |
|  |  | *Nebria* sp.n. 2 near *orestias* | 4200-(4500 m: r) | 28.351395 | 84.739129 | 8,41 | 26,03 |
| 6 | NEPAL, western Helambu Massif, | *Amara nepalensis* | 4000-(4450 m: ?) | 28.060851 | 85.450233 | 8,99 | 24,22 |
|  | around Gosainkundh |  |  |  |  |  |  |
| 7 | NEPAL, central Helambu Massif, | *Amara nepalensis* | 4200-4600 m | 28.127498 | 85.592379 | 8,57 | 27,09 |
|  | S-slope of Kanja La |  |  |  |  |  |  |
| 8 | NEPAL, eastern Helambu Massif, | *Amara* sp.n. near *nepalensis* | 4100-(4350 m: ?) | 27.986537 | 85.881513 | 7,20 | 26,28 |
|  | Bhairav Kundh |  |  |  |  |  |  |
| 9 | NEPAL, Solu Khumbu Massif, | *Amara* sp.n. 1 near *khumbuensis* | 4200-(4600 m: r) | 27.685652 | 86.588962 | 7,40 | 26,74 |
|  | Dudh Kundh | *Nebria* sp.n. 3 near *orestias* | 4300-4500 m | 27.689647 | 86.589189 | 6,84 | 26,98 |
| 10 | NEPAL, Kalo Himal, | *Amara* sp.n. 2 near *khumbuensis* | 4400-(4600 m: t) | 27.644874 | 86.774193 | 4,97 | 26,07 |
|  | Zatrwa La |  |  |  |  |  |  |
| **Greater Himalaya main ridge** | | | | | | | |
| 11 | NEPAL, E-slope Dhaulagiri I, | *Amara thorongiensis* | 4000-(4200 m: r) | 28.684122 | 83.561009 | 10,53 | 25,60 |
|  | Dhaulagiri E-glacier |  |  |  |  |  |  |
| 12 | NEPAL, W-slope Nilgiri Himal, | *Amara thorongiensis* | 4200-(4500 m: r) | 28.679757 | 83.683834 | 8,24 | 26,75 |
|  | Marchhe Lekh | *Deltomerodes* sp.n. 1 near *chulii* | 4300-(4500 m: r) | 28.678016 | 83.686235 | 7,69 | 26,84 |
|  |  | *Trechus breuningi* | 4200-(4500 m: r) | 28.679757 | 83.683834 | 8,24 | 26,75 |
| 13 | NEPAL, S-slope Nilgiri Himal, | *Amara* sp.n. 3 near *jaljalensis* | 4000-4600 m | 28.612965 | 83.681507 | 8,44 | 27,97 |
|  | Thulo Bugin | *Amara thorongiensis* | 4200-4600 m | 28.612855 | 83.683762 | 7,24 | 28,14 |
|  |  | *Trechus breuningi* | 4200-4600 m | 28.612855 | 83.683762 | 7,24 | 28,14 |
| 14 | NEPAL, W-slope Annapurna South, | *Amara* sp.n. 2 near *franzi* | 4300-(4700 m) | 28.497533 | 83.745531 | 6,10 | 27,23 |
|  | Khayer Lake | *Deltomerodes* sp.n. 1 near *chulii* | 4500-(4700 m) | 28.499503 | 83.750570 | 4,84 | 27,63 |
|  |  | *Nebria* sp.n. 4 near *orestias* | 4500-(4600 m) | 28.499503 | 83.750570 | 4,84 | 27,63 |
|  |  | *Trechus* sp.n. 2 near *breuningi* | 4300-(4700 m) | 28.497533 | 83.745531 | 6,10 | 27,23 |
| 15 | NEPAL, S-slope Lamjung Himal, | *Deltomerodes nepalensis* | 4500-(4650 m: r) | 28.455641 | 84.265263 | 6,00 | 27,56 |
|  | around Dudh Pokhari | *Trechus gurungi* | 4450-(4650 m: r) | 28.454428 | 84.268308 | 6,35 | 27,33 |
| 16 | NEPAL, E-slope Lamjung Himal, | *Deltomerodes nepalensis* | 4400-(4500 m: r) | 28.474934 | 84.307282 | 6,45 | 26,03 |
|  | N-slope Namun La | *Deltomerodes* sp.n. 2 near *chulii* | 4400-(4500 m: r) | 28.474934 | 84.307282 | 6,45 | 26,03 |
|  |  | *Trechus gurungi* | 4400-(4500 m: r) | 28.474934 | 84.307282 | 6,45 | 26,03 |
| 17 | NEPAL, N-slope Rolwaling Vall., | *Nebria molendai* | 5000-(5050 m: r) | 27.907457 | 86.468488 | 3,12 | 29,98 |
|  | Omei Tsho |  |  |  |  |  |  |
| 18 | NEPAL, S-slope Rolwaling Vall., | *Deltomerodes* sp.n. 1 near *sciakyi* | 4550-(4650 m: r) | 27.865773 | 86.465182 | 5,91 | 29,63 |
|  | Tsho Rolpa | *Nebria molendai* | 4550-(4650 m: r) | 27.865773 | 86.465182 | 5,91 | 29,63 |
| 19 | NEPAL, S-slope Rolwaling Vall., | *Deltomerodes* sp.n. 1 near *sciakyi* | 4600-(5050 m: r) | 27.868056 | 86.444764 | 5,97 | 29,07 |
|  | Yarlung Ri base camp | *Nebria christinae* | 4600-5000 m | 27.868056 | 86.444764 | 5,97 | 29,07 |
|  |  | *Nebria molendai* | 4600-5000 m | 27.868056 | 86.444764 | 5,97 | 29,07 |
| 20 | NEPAL, S-slope Hunku Himal, | *Amara* sp.n. 3 near *khumbuensis* | 4900-(5150 m: r) | 27.745108 | 86.877099 | 2,73 | 30,86 |
|  | Khare Glacier moraine | *Deltomerodes* sp.n. 2 near *sciakyi* | 4950-(5150 m: r) | 27.747261 | 86.878599 | 2,44 | 30,91 |
|  |  | *Trechus* sp.n. 1 near *morvanianus* | 4950-(5150 m: r) | 27.747261 | 86.878599 | 2,44 | 30,91 |
| 21 | NEPAL, upper Barun Valley, | *Amara sankhuana* | 4700-(4900 m: t) | 27.807569 | 87.078598 | 5,78 | 28,84 |
|  | Barun Glacier moraine | *Deltomerodes* sp.n. near *stenomus* | 4800-(4900 m: t) | 27.813938 | 87.075582 | 5,12 | 29,20 |
|  |  | *Trechus* sp.n. 2 near *morvanianus* | 4750-(4900 m: t) | 27.809283 | 87.075233 | 5,46 | 28,95 |
| 22 | NEPAL, upper Barun Valley, | *Amara sankhuana* | 4700-(5250 m: r) | 27.805138 | 87.092337 | 5,75 | 29,01 |
|  | above Shershong | *Deltomerodes* sp.n. near *stenomus* | 5000-(5250 m: r) | 27.815936 | 87.085701 | 3,95 | 29,75 |
|  |  | *Trechus* sp.n. 2 near *morvanianus* | 5000-(5250 m: r) | 27.815936 | 87.085701 | 3,95 | 29,75 |
| 23 | NEPAL, upper Barun Valley, | *Amara sankhuana* | 4450-(4750 m: r) | 27.797662 | 87.126524 | 7,11 | 28,60 |
|  | above Langmale Kharka | *Deltomerodes* sp.n. near *stenomus* | 4700-(4750 m: r) | 27.806979 | 87.127050 | 5,79 | 28,76 |
|  |  | *Trechus* sp.n. 2 near *morvanianus* | 4700-(4750 m: r) | 27.806979 | 87.127050 | 5,79 | 28,76 |
| 24 | NEPAL, Lumbasumba Himal, | *Amara sankhuana* | 4300-(4650 m: t) | 27.662026 | 87.593721 | 7,39 | 26,24 |
|  | arround Thangla Bhanjyang | *Amara schawalleri* | 4300-(4650 m: t) | 27.662026 | 87.593721 | 7,39 | 26,24 |
|  |  | *Deltomerodes schawalleri* | 4600-(4650 m: t) | 27.673767 | 87.589031 | 5,56 | 27,03 |
| 25 | NEPAL, Lumbasumba Himal, | *Amara sankhuana* | 4150-(4850 m: r) | 27.756608 | 87.590628 | 8,93 | 27,25 |
|  | W-slope Lumbasumba pass | *Amara schawalleri* | 4150-(4850 m: r) | 27.756608 | 87.590628 | 8,93 | 27,25 |
|  |  | *Deltomerodes schawalleri* | 4600-(4850 m: r) | 27.744586 | 87.590514 | 6,09 | 27,70 |
|  |  | *Deltomerodes* sp.n. near *stenomus* | 4600-(4850 m: r) | 27.744586 | 87.590514 | 6,09 | 27,70 |
| 26 | NEPAL, Lumbasumba Himal, | *Amara sankhuana* | 4150-(4750 m: r) | 27.717991 | 87.702361 | 8,77 | 27,54 |
|  | S-slope Lumbasumba pass | *Amara schawalleri* | 4150-(4750 m: r) | 27.717991 | 87.702361 | 8,77 | 27,54 |
|  |  | *Amara* sp.n. near *jannui* | 4150-(4750 m: r) | 27.717991 | 87.702361 | 8,77 | 27,54 |
|  |  | *Deltomerodes schawalleri* | 4600-(4750 m: r) | 27.739629 | 87.682588 | 6,36 | 28,48 |
| **Greater Himalaya north slope** | | | | | | | |
| 27 | NEPAL, NW-slope Dhaulagiri I, | *Amara martensi* | 4700-(4800 m: r) | 28.750039 | 83.500200 | 6,90 | 29,37 |
|  | Dhaulagiri base camp |  |  |  |  |  |  |
| 28 | N-slope Dhaulagiri I, | *Amara martensi* | 4700-5100 m | 28.857241 | 83.584793 | 8,24 | 27,98 |
|  | around Hidden Valley | *Bembidion pluto* | 4700-5100 m | 28.857241 | 83.584793 | 8,24 | 27,98 |
|  |  | *Nebria superna* | 4850-5150 m | 28.825543 | 83.584550 | 6,81 | 29,26 |
| 29 | NEPAL, NE-slope Dhaulagiri I, | *Amara thorongiensis* | 4200-4550 m | 28.747477 | 83.634674 | 9,50 | 26,72 |
|  | above Yakkharka | *Deltomerodes nepalensis* | 4600-4800 m | 28.753719 | 83.634199 | 6,91 | 27,46 |
|  |  | *Deltomerodes* sp.n. 1 near *chulii* | 4600-4800 m | 28.753719 | 83.634199 | 6,91 | 27,46 |
|  |  | *Trechus tilitshoensis* | 4600-4800 m | 28.753719 | 83.634199 | 6,91 | 27,46 |
| 30 | NEPAL, W-slope Muktinath Himal, | *Amara thorongiensis* | 4350-(4500 m: ?) | 28.797997 | 83.875849 | 8,71 | 27,58 |
|  | south above Muktinath |  |  |  |  |  |  |
| 31 | NEPAL, W-slope Muktinath Himal, | *Amara thorongiensis* | 4500-5000 m | 28.809039 | 83.900577 | 8,15 | 28,30 |
|  | W-slope Thorong La |  |  |  |  |  |  |
| 32 | NEPAL, E-slope Muktinath Himal, | *Amara thorongiensis* | 4900-5150 m | 28.783752 | 83.966446 | 6,25 | 28,47 |
|  | E-slope Thorong La | *Deltomerodes nepalensis* | 5000-5150 m | 28.785363 | 83.961194 | 5,50 | 28,66 |
|  |  | *Trechus thorongiensis* | 4900-(4950 m: ?) | 28.783752 | 83.966446 | 6,25 | 28,47 |
|  |  | *Trechus tilitshoensis* | 5000-5150 m | 28.785363 | 83.961194 | 5,50 | 28,66 |
| 33 | NEPAL, NW-slope Chulu Himal, | *Amara thorongiensis* | 4700-4950 m | 28.769364 | 83.975826 | 7,45 | 28,48 |
|  | east above Thorung Phedi | *Deltomerodes nepalensis* | 4800-(5050 m: r) | 28.765810 | 83.977152 | 6,75 | 28,80 |
|  |  | *Trechus tilitshoensis* | 4700-4950 m | 28.769364 | 83.975826 | 7,45 | 28,48 |
| 34 | NEPAL, SE-slope Chulu Himal, | *Deltomerodes nepalensis* | 4950-(5050 m: r) | 28.686722 | 84.137718 | 4,70 | 28,12 |
|  | E-slope Kangla | *Trechus tilitshoensis* | 4950-(5050 m: r) | 28.686722 | 84.137718 | 4,70 | 28,12 |
| 35 | NEPAL, N-slope Pisang Peak, | *Amara pisangana* | 4750-4900 m | 28.667348 | 84.186718 | 5,13 | 29,03 |
|  | above Naar | *Deltomerodes nepalensis* | 4750-4900 m | 28.667348 | 84.186718 | 5,13 | 29,03 |
| 36 | NEPAL, N-slope Annapurna Himal, | *Amara schmidti* | 4900-5050 m | 28.680420 | 83.865838 | 5,75 | 29,11 |
|  | around Tilitsho | *Deltomerodes nepalensis* | 4900-5050 m | 28.680420 | 83.865838 | 5,75 | 29,11 |
|  |  | *Trechus tilitshoensis* | 4900-5050 m | 28.680420 | 83.865838 | 5,75 | 29,11 |
| **Tibetan Himalaya** | | | | | | | |
| 37 | CHINA, S-Tibet,Tibetan Himalaya, | *Amara brucei* | 5000-(5150 m: t) | 28.508544 | 86.166047 | 5,91 | 29,02 |
|  | around Tong La | *Amara everesti* | 5000-(5150 m: t) | 28.508544 | 86.166047 | 5,91 | 29,02 |
|  |  | *Amara hypsela* | 5000-(5150 m: t) | 28.508544 | 86.166047 | 5,91 | 29,02 |
| 38 | CHINA, S-Tibet,Tibetan Himalaya, | *Amara sachiana* | 4900-(5350 m: r) | 28.787988 | 87.936050 | 7,78 | 29,60 |
|  | around Dongo La | *Casaleianus ferrugineus* | 4900-5200 m | 28.787988 | 87.936050 | 7,78 | 29,60 |
|  |  | *Trechus dongola* | 5100-(5350 m: r) | 28.788436 | 87.942670 | 6,59 | 30,00 |
|  |  | *Trechus thibetanus* | 4900-5200 m | 28.787988 | 87.936050 | 7,78 | 29,60 |
| 39 | CHINA, S-Tibet,Tibetan Himalaya, | *Amara lhatsensis* | 5000-(5350 m: ?) | 28.973454 | 87.459552 | 8,07 | 29,56 |
|  | around Gyatso La | *Casaleianus* sp.n. 1 near *ferrugineus* | 5150-5250 m | 28.959313 | 87.423514 | 7,06 | 29,57 |
|  |  | *Nebria superna* | 5200-(5350 m: ?) | 28.956068 | 87.416961 | 6,76 | 29,54 |
|  |  | *Trechus gyatsola* | 5200-(5350 m: ?) | 28.956068 | 87.416961 | 6,76 | 29,54 |
|  |  | *Trechus thibetanus* | 5000-5300 m | 28.973454 | 87.459552 | 8,07 | 29,56 |
| 40 | CHINA, S-Tibet,Tibetan Himalaya, | *Amara lhatsensis* | 4700-(4950 m: t) | 29.066490 | 87.983986 | 8,44 | 29,09 |
|  | around Tsuo La |  |  |  |  |  |  |
| 41 | CHINA, S-Tibet,Tibetan Himalaya, | *Amara karolana* | 4800-5400 m | 28.967574 | 89.815793 | 7,48 | 28,97 |
|  | around Yung La | *Amara karolanella* | 4750-5400 m | 28.969654 | 89.815186 | 7,78 | 28,92 |
|  |  | *Trechus thibetanus* | 4850-5400 m | 28.965576 | 89.816616 | 7,17 | 29,01 |
| 42 | CHINA, S-Tibet,Tibetan Himalaya, | *Amara karolana* | 4750-(5300 m: r) | 28.900085 | 90.296615 | 9,48 | 28,94 |
|  | E side of Karo La | *Amara karolanella* | 4750-(5300 m: r) | 28.900085 | 90.296615 | 9,48 | 28,94 |
|  |  | *Bembidion* sp.n. near *shugela* | 4900-5000 m | 28.909258 | 90.300134 | 8,56 | 28,41 |
|  |  | *Casaleianus* sp.n. 2 near *ferrugineus* | 4900-5000 m | 28.909258 | 90.300134 | 8,56 | 28,41 |
|  |  | *Nebria superna* | 4950-5200 m | 28.911623 | 90.302152 | 8,16 | 28,42 |
|  |  | *Trechus thibetanus* | 4850-(5300 m: r) | 28.905282 | 90.299201 | 8,89 | 28,64 |
| 43 | CHINA, S-Tibet,Tibetan Himalaya, | *Amara kampalaensis* | 4750-(4900 m: t) | 29.198709 | 90.611580 | 8,53 | 28,69 |
|  | N side of Kampa La | *Casaleianus* sp.n. 2 near *ferrugineus* | 4800-(4900 m: t) | 29.195928 | 90.614452 | 8,27 | 28,85 |
|  |  | *Trechus thibetanus* | 4750-(4900 m: t) | 29.198709 | 90.611580 | 8,53 | 28,69 |
| 44 | CHINA, S-Tibet,Tibetan Himalaya, | *Amara lhozhagensis* | 5000-(5200 m: ?) | 28.626193 | 90.445557 | 6,68 | 29,27 |
|  | N-slope Monda La | *Amara karolana* | 5000-(5100 m) | 28.652619 | 90.444353 | 6,81 | 29,13 |
|  |  | *Bembidion pluto* | 5050-(5150 m) | 28.493033 | 90.547807 | 6,50 | 29,53 |
|  |  | *Nebria superna* | 5050-(5150 m) | 28.493033 | 90.547807 | 6,50 | 29,53 |
|  |  | *Trechus eutrechoides* | 5050-(5150 m) | 28.493033 | 90.547807 | 6,50 | 29,53 |
|  |  | *Trechus pumoensis* | 5050-(5150 m) | 28.493033 | 90.547807 | 6,50 | 29,53 |
|  |  | *Trechus thibetanus* | 4800-(5100 m) | 28.642690 | 90.441648 | 8,04 | 29,22 |
| **Transhimalaya (Gangdise Shan)** | | | | | | | |
| 45 | CHINA, S-Tibet, Gangdise Shan, | *Amara* sp.n. 1 near *kangtissuensis* | 4800-(5300 m: r) | 29.508741 | 90.682378 | 8,58 | 28,43 |
|  | Chusul Valley, Rana side valley | *Apatrobus* sp.n. 1 | 5200-(5300 m: r) | 29.515827 | 90.674514 | 6,06 | 29,12 |
|  |  | *Trechus hiekei hiekei* | 5100-(5300 m: r) | 29.510839 | 90.677717 | 6,64 | 29,20 |
|  |  | *Trechus thibetanus* | 5000-(5300 m: r) | 29.510374 | 90.679353 | 7,29 | 28,93 |
| 46 | CHINA, S-Tibet, Gangdise Shan, | *Amara* sp.n. 1 near *kangtissuensis* | 4850-(5000 m: ?) | 29.493016 | 90.739268 | 7,72 | 28,96 |
|  | Chusul Valley, Baypama side vall. | *Trechus thibetanus* | 4850-(5000 m: ?) | 29.493016 | 90.739268 | 7,72 | 28,96 |
| 47 | CHINA, S-Tibet, Gangdise Shan, | *Amara* sp.n. 2 near *kangtissuensis* | 4900-(5350 m: r) | 29.681625 | 90.758011 | 7,64 | 29,25 |
|  | Kurum Valley,Namba side valley | *Apatrobus* sp.n. 1 | 4900-5300 m | 29.681625 | 90.758011 | 7,64 | 29,25 |
|  |  | *Deltomerodes* sp.n. 1 near *wrasei* | 5100-(5350 m: r) | 29.676753 | 90.764673 | 6,06 | 29,75 |
|  |  | *Nebria mentoincisa* | 5100-(5350 m: r) | 29.676753 | 90.764673 | 6,06 | 29,75 |
|  |  | *Trechus hiekei subtiliformis* | 5100-(5350 m: r) | 29.676753 | 90.764673 | 6,06 | 29,75 |
|  |  | *Trechus tsampa tsampa* | 5100-(5350 m: r) | 29.676753 | 90.764673 | 6,06 | 29,75 |
| 48 | CHINA, S-Tibet, Gangdise Shan, | *Amara* sp.n. 3 near *kangtissuensis* | 4900-(5450 m: r) | 29.763114 | 90.698836 | 7,84 | 28,07 |
|  | Kurum Vall., above Nenang Mon. | *Apatrobus* sp.n. 1 | 5100-(5450 m: r) | 29.765776 | 90.695459 | 6,48 | 28,45 |
| 49 | CHINA, S-Tibet, Gangdise Shan, | *Amara* sp.n. 4 near *kangtissuensis* | 4900-(5200 m: ?) | 29.712848 | 90.587617 | 7,80 | 29,70 |
|  | Kurum Vall., above Tsurphu Mon. | *Apatrobus* sp.n. 1 | 5100-(5200 m: ?) | 29.707810 | 90.589360 | 6,41 | 29,93 |
|  |  | *Deltomerodes* sp.n. 1 near *wrasei* | 5150-(5200 m: ?) | 29.706646 | 90.589941 | 6,07 | 30,01 |
|  |  | *Nebria mentoincisa* | 5000-(5200 m: ?) | 29.710301 | 90.588585 | 7,11 | 29,81 |
|  |  | *Nebria superna* | 5000-(5200 m: ?) | 29.710301 | 90.588585 | 7,11 | 29,81 |
|  |  | *Trechus lama* | 4900-(5200 m: ?) | 29.712848 | 90.587617 | 7,80 | 29,70 |
| 50 | CHINA, S-Tibet, Gangdise Shan, | *Apatrobus* sp.n. 2 | 5050-(5150 m: ?) | 29.718514 | 91.030029 | 5,63 | 28,65 |
|  | Mt. Utsi N of Lhasa Valley | *Trechus utsi* | 5050-(5150 m: ?) | 29.718514 | 91.030029 | 5,63 | 28,65 |
| 51 | CHINA, S-Tibet, Gangdise Shan, | *Amara* sp.n. 1 near *heinzorum* | 4700-5400 m | 29.579276 | 91.225406 | 8,56 | 29,38 |
|  | Mt. Mendju Zari S of Lhasa Valley | *Deltomerodes wrasei* | 5300-5400 m | 29.573428 | 91.235272 | 4,68 | 30,27 |
|  |  | *Nebria* sp.n. near *businskyorum* | 5300-5400 m | 29.573428 | 91.235272 | 4,68 | 30,27 |
|  |  | *Trechus lhasaensis* | 4700-(5450 m: t) | 29.579276 | 91.225406 | 8,56 | 29,38 |
| 52 | CHINA, S-Tibet, Gangdise Shan, | *Amara* sp.n. 2 near *heinzorum* | 4900-(5250 m: r) | 29.682746 | 91.593637 | 7,03 | 28,80 |
|  | upper Gyama Valley | *Apatrobus* sp.n. 3 | 5150-5200 m | 29.674458 | 91.582033 | 5,36 | 29,61 |
|  |  | *Deltomerodes wrasei* | 5150-(5250 m: r) | 29.674458 | 91.582033 | 5,36 | 29,61 |
|  |  | *Trechus martinae* | 5150-(5250 m: r) | 29.674458 | 91.582033 | 5,36 | 29,61 |
| 53 | CHINA, S-Tibet, Gangdise Shan, | *Trechus korae* | 5150-(5250 m: t) | 29.681719 | 92.204526 | 4,31 | 30,26 |
|  | mountain 2km SW Rutok |  |  |  |  |  |  |
| 54 | CHINA, S-Tibet, Gangdise Shan, | *Apatrobus* sp.n. 4 | 5000-(5200 m: t) | 29.824333 | 92.339809 | 5,43 | 29,15 |
|  | around Mila | *Deltomerodes* sp.n. 2 near *wrasei* | 5100-(5200 m: t) | 29.828308 | 92.334775 | 4,68 | 29,65 |
|  |  | *Trechus mila* | 5000-(5200 m: t) | 29.824333 | 92.339809 | 5,43 | 29,15 |
| 55 | CHINA, S-Tibet, Gangdise Shan, | *Amara* sp.n. 3 near *heinzorum* | 4800-(5350 m: t) | 30.120665 | 92.203299 | 7,14 | 28,28 |
|  | Mt. Nede Chuto N Menpa | *Apatrobus* sp.n. 4 | 5200-(5350 m: t) | 30.130639 | 92.204875 | 3,95 | 29,30 |
|  |  | Trechus menpa | 4800-(5350 m: t) | 30.120665 | 92.203299 | 7,14 | 28,28 |
| 56 | CHINA, S-Tibet, Gangdise Shan, | *Amara* sp.n. 1 near *carexiphaga* | 4900-(5250 m: t) | 30.323898 | 91.508913 | 6,84 | 28,29 |
|  | north above Reting Monastery |  |  |  |  |  |  |
| 57 | CHINA, S-Tibet, Gangdise Shan, | *Trechus mieheorum* | 5150-(5200 m: r) | 30.408192 | 91.688241 | 4,80 | 28,69 |
|  | upper Kiykiy valley E Reting |  |  |  |  |  |  |
| 58 | CHINA, S-Tibet, Gangdise Shan, | *Amara* sp.n. near *chaklaensis* | 5000-(5200 m: r) | 30.117322 | 91.271917 | 5,84 | 29,27 |
|  | Lhundup, above Chak La | *Trechus wrzecionkoianus* | 5000-(5200 m: r) | 30.117322 | 91.271917 | 5,84 | 29,27 |
| 59 | CHINA, S-Tibet, Gangdise Shan, | *Amara* sp.n. 2 near *carexiphaga* | 4900-(5200 m: ?) | 30.236768 | 91.038508 | 6,52 | 29,19 |
|  | upper Nejam Tsu Valley | *Trechus neyamensis* | 4900-(5050 m: ?) | 30.236768 | 91.038508 | 6,52 | 29,19 |
|  |  | *Trechus parvifrater* | 4900-(5050 m: ?) | 30.236768 | 91.038508 | 6,52 | 29,19 |
| 60 | CHINA, S-Tibet, Gangdise Shan, | *Amara altiphila* | 5000-(5400 m: t) | 30.129661 | 90.593620 | 6,19 | 29,65 |
|  | above Shemalung Monastery | *Bembidion maddisoni* | 4900-(5400 m: t) | 30.130097 | 90.588683 | 6,79 | 29,60 |
|  |  | *Trechus christinae* | 4950-(5400 m: t) | 30.129209 | 90.591061 | 6,48 | 29,62 |
| 61 | CHINA, S-Tibet, Gangdise Shan, | *Amara* sp.n. 3 near *carexiphaga* | 4850-(5150 m: r) | 30.053817 | 90.535382 | 7,83 | 29,54 |
|  | N-slope Mt. Birze | *Trechus religiosus birze* | 4850-(5150 m: r) | 30.053817 | 90.535382 | 7,83 | 29,54 |
| 62 | CHINA, S-Tibet, Gangdise Shan, | *Amara* sp.n. 1 near *shogulaensis* | 5000-(5350 m: r) | 29.949536 | 90.507515 | 7,31 | 29,48 |
|  | W-slope Mt. Birze | *Apatrobus* sp.n. 1 | 5200-(5350 m: r) | 29.954585 | 90.511603 | 5,84 | 29,92 |
|  |  | *Nebria superna* | 5200-(5350 m: r) | 29.954585 | 90.511603 | 5,84 | 29,92 |
|  |  | *Bembidion maddisoni* | 5100-(5350 m: r) | 29.957347 | 90.507063 | 6,66 | 29,67 |
|  |  | *Trechus tsampa striatopunctatus* | 5000-(5350 m: r) | 29.949536 | 90.507515 | 7,31 | 29,48 |
| 63 | CHINA, S-Tibet, | *Amara shogulaensis* | 5000-5500 m | 29.769051 | 90.300908 | 7,16 | 29,20 |
|  | S-face Nyainqentanglha Shan, | *Amara* sp.n. 3 near *carexiphaga* | 5000-(5550 m: r) | 29.769051 | 90.300908 | 7,16 | 29,20 |
|  | eastern morain of Mt. Pijin | *Amara* sp.n. 1 near *yanpachensis* | 5000-(5550 m: r) | 29.769051 | 90.300908 | 7,16 | 29,20 |
|  |  | *Bembidion maddisoni* | 5400-5500 m | 29.761449 | 90.301917 | 4,54 | 30,14 |
|  |  | *Nebria superna* | 5100-5200 m | 29.766822 | 90.300510 | 6,51 | 29,44 |
|  |  | *Trechus gyalpo* | 4950-5400 m | 29.769783 | 90.303704 | 7,49 | 29,13 |
|  |  | *Trechus folwarcznyi* | 5000-5500 m | 29.769051 | 90.300908 | 7,16 | 29,20 |
| 64 | CHINA, S-Tibet, | *Amara yangpachensis* | 5150-(5450 m: t) | 29.919172 | 90.124544 | 6,65 | 29,88 |
|  | S-face Nyainqentanglha Shan, | *Bembidion maddisoni* | 5000-(5450 m: t) | 29.921177 | 90.151971 | 7,76 | 29,97 |
|  | around Shogu La | *Nebria superna* | 5100-5200 m | 29.921593 | 90.130822 | 7,07 | 29,91 |
|  |  | *Nebria walteriana* | 5100-5350 m | 29.921593 | 90.130822 | 7,07 | 29,91 |
|  |  | *Trechus bastropi* | 5250-5350 m | 29.915909 | 90.125272 | 5,99 | 29,98 |
|  |  | *Trechus folwarcznyi* | 5100-(5450 m: t) | 29.921593 | 90.130822 | 7,07 | 29,91 |
|  |  | *Trechus yak shogulaensis* | 5000-5350 m | 29.921177 | 90.151971 | 7,76 | 29,97 |
|  |  | *Trechus yeti* | 5100-5300 m | 29.921593 | 90.130822 | 7,07 | 29,91 |
| 65 | CHINA, S-Tibet, | *Amara altiphila* | 5100-(5300 m: ?) | 30.097682 | 90.352905 | 6,27 | 28,78 |
|  | S-face Nyainqentanglha Shan, | *Bembidion maddisoni* | 5050-(5300 m: ?) | 30.085190 | 90.358654 | 6,56 | 28,83 |
|  | upper Linchung Valley | *Nebria superna* | 5150-5250 m | 30.104987 | 90.346781 | 5,97 | 28,96 |
|  |  | *Trechus budhaensis linchungensis* | 5150-(5300 m: ?) | 30.104987 | 90.346781 | 5,97 | 28,96 |
|  |  | *Trechus yak shogulaensis* | 5050-(5300 m: ?) | 30.085190 | 90.358654 | 6,56 | 28,83 |
| 66 | CHINA, S-Tibet, | *Amara altiphila* | 5100-(5350 m: ?) | 30.117605 | 90.452261 | 6,12 | 28,36 |
|  | S-face Nyainqentanglha Shan, | *Bembidion maddisoni* | 5000-(5350 m: ?) | 30.113161 | 90.451780 | 6,80 | 28,23 |
|  | mountain N Yangpachem | *Nebria superna* | 5150-(5350 m: ?) | 30.119545 | 90.450021 | 5,79 | 28,39 |
|  |  | *Trechus budhaensis* | 5000-5300 m | 30.113161 | 90.451780 | 6,80 | 28,23 |
|  |  | *Trechus yak yak* | 5150-(5350 m: ?) | 30.119545 | 90.450021 | 5,79 | 28,39 |
| 67 | CHINA, S-Tibet, | *Amara altiphila* | 5100-(5600 m: r) | 30.184186 | 90.486340 | 6,13 | 28,60 |
|  | S-face Nyainqentanglha Shan, | *Bembidion maddisoni* | 5000-(5600 m: r) | 30.182148 | 90.490150 | 6,79 | 28,41 |
|  | upper Budha Valley | *Nebria superna* | 5200-5300 m | 30.183669 | 90.481194 | 5,55 | 28,75 |
|  |  | *Trechus astrophilus* | 5200-(5600 m: r) | 30.183669 | 90.481194 | 5,55 | 28,75 |
|  |  | *Trechus budhaensis* | 5200-5500 m | 30.183669 | 90.481194 | 5,55 | 28,75 |
|  |  | *Trechus yak yak* | 5000-5450 m | 30.182148 | 90.490150 | 6,79 | 28,41 |
| 68 | CHINA, S-Tibet, | *Amara altiphila* | 5100-(5400 m: r) | 30.221415 | 90.529316 | 6,06 | 29,27 |
|  | S-face Nyainqentanglha Shan, | *Bembidion maddisoni* | 5100-(5400 m: r) | 30.221415 | 90.529316 | 6,06 | 29,27 |
|  | upper Lombuk Tsu Valley | *Nebria superna* | 5150-5250 m | 30.223715 | 90.528241 | 5,77 | 29,37 |
|  |  | *Trechus religiosus nigropiceus* | 4900-(5400 m: r) | 30.210593 | 90.532064 | 7,33 | 28,76 |
| 69 | CHINA, S-Tibet, | *Amara altiphila* | 5200-(5400 m: r) | 30.305367 | 90.621747 | 5,52 | 29,11 |
|  | S-face Nyainqentanglha Shan, | *Bembidion maddisoni* | 5200-(5400 m: r) | 30.305367 | 90.621747 | 5,52 | 29,11 |
|  | SW Nyainqentanglha Feng | *Trechus religiosus religiosus* | 4900-(5500 m: r) | 30.294589 | 90.612802 | 7,45 | 28,90 |
| 70 | CHINA, S-Tibet, | *Amara* sp.n. near *altiphila* | 5200-(5350 m: r) | 30.378097 | 90.725696 | 5,23 | 29,04 |
|  | S-face Nyainqentanglha Shan, | *Bembidion maddisoni* | 5250-(5350 m: r) | 30.379053 | 90.723688 | 4,88 | 29,23 |
|  | Lha Tsu Valley | *Trechus religiosus lhai* | 4900-(5350 m: r) | 30.367293 | 90.723764 | 7,21 | 28,75 |
|  |  | *Trechus opgenoorthi* | 5000-(5350 m: r) | 30.371414 | 90.724989 | 6,58 | 28,81 |
| 71 | CHINA, S-Tibet, | *Amara* sp.n. near *altiphila* | 5200-(5300 m: ?) | 30.636161 | 91.102242 | 4,17 | 29,85 |
|  | S-face Nyainqentanglha Shan, | *Trechus antonini* | 5100-(5300 m: ?) | 30.638670 | 91.103033 | 4,83 | 29,67 |
|  | around Lhachen La | *Trechus hodeberti* | 5100-5150 m | 30.638670 | 91.103033 | 4,83 | 29,67 |
| 72 | CHINA, S-Tibet, | *Amara* sp.n. near *altiphila* | 5100-(5200 m: r) | 30.628920 | 90.869682 | 5,85 | 30,03 |
|  | N-face Nyainqentanglha Shan, | *Trechus antonini* | 5150-(5200 m: r) | 30.627976 | 90.870822 | 5,52 | 30,13 |
|  | Langma Valley |  |  |  |  |  |  |
| 73 | CHINA, S-Tibet, | *Amara* sp.n. 4 near *carexiphaga* | 5150-(5400 m: r) | 30.572205 | 90.750499 | 5,49 | 30,29 |
|  | N-face Nyainqentanglha Shan, | *Trechus nami* | 5100-(5400 m: r) | 30.574757 | 90.749179 | 5,87 | 30,14 |
|  | Shashe Tsu Valley | *Nebria superna* | 5100-5200 m | 30.574757 | 90.749179 | 5,87 | 30,14 |
| 74 | CHINA, S-Tibet, Gangdise Shan, | *Amara brucei* | 4900-(5200 m: t) | 29.486921 | 86.419088 | 9,35 | 29,54 |
|  | Sang Sang, Pel La | *Amara* sp.n. near *karolana* | 4900-(5200 m: t) | 29.486921 | 86.419088 | 9,35 | 29,54 |
|  |  | *Amara* sp.n. 2 near *shogulaensis* | 4950-(5200 m: t) | 29.485754 | 86.417979 | 9,00 | 29,64 |
|  |  | *Casaleianus* sp.n. 3 near *ferrugineus* | 4950-5100 m | 29.485754 | 86.417979 | 9,00 | 29,64 |
| 75 | CHINA, S-Tibet, Gangdise Shan, | *Amara brucei* | 4950-(5350 m: r) | 29.539325 | 86.310983 | 8,72 | 29,41 |
|  | Mt. Syu | *Amara* sp.n. near *karolana* | 4900-(5350 m: r) | 29.535604 | 86.307701 | 9,11 | 29,32 |
|  |  | *Amara* sp.n. 2 near *shogulaensis* | 4950-(5350 m: r) | 29.539325 | 86.310983 | 8,72 | 29,41 |
|  |  | *Casaleianus* sp.n. 3 near *ferrugineus* | 5150-5300 m | 29.542614 | 86.326362 | 7,23 | 29,77 |
|  |  | *Nebria superna* | 5200-5300 m | 29.542396 | 86.329572 | 6,88 | 29,81 |
| 76 | CHINA, S-Tibet, Gangdise Shan, | *Amara* sp.n. near *karolana* | 4900-(5350 m: r) | 30.419943 | 86.490574 | 9,75 | 29,00 |
|  | S-slope Mt. Lungmari | *Amara* sp.n. 2 near *shogulaensis* | 5000-(5350 m: r) | 30.423823 | 86.486779 | 9,06 | 29,03 |
|  |  | *Amara* sp.n. 2 near *yanpachensis* | 5000-(5350 m: r) | 30.423823 | 86.486779 | 9,06 | 29,03 |
|  |  | *Nebria superna* | 5100-5300 m | 30.424109 | 86.483256 | 8,38 | 29,13 |

* According to the method used by Schmidt et al. (2011) the values of the upper and lower range limits were rounded up and down respectively, to 50 m steps. This method takes account of the fact of the limited accuracy attainable during field work. Data in brackets: the actual upper distributional limits are uncertain because: (r) the upper part of the mountain slope is covered by thick layers of scree and thus is unsuitable as ground beetle habitat; (t) the species were found up to the local mountain crest; (?) the data were insufficiently proofed in the field.

**Coordinates are given for the lowest occurrences of the respective species observed in the field.
