# Supplementary material for: Mass elevation and lee effects markedly lift the elevational distribution of ground beetles in the Himalaya-Tibet orogen
Source: PLoS One. 2017 Mar 24;12(3):e0172939. doi: 10.1371/journal.pone.0172939 (PMC5365098; doi:10.1371/journal.pone.0172939)
Supplement: S2 Table — Comparison of random effect structures of models with temperature that either (A) included species (N = 118) or (B) excluded species (N = 232). (DOCX) [file pone.0172939.s002.docx]

**Table S2** Comparison of random effect structures for models that either (A) included species (*N* = 118) or (B) excluded species (*N* = 232)

| Model | Marginal R² | Conditional R² | AIC |
| --- | --- | --- | --- |
| (A) Species included | | | |
| lmer(elevation ~ temp.july + location + temp.july * location + (1\|Carabidae_species)) | 0.90 | 0.94 | 2782 |
| lmer(elevation ~ temp.july + location + (1\|Carabidae_species)) | 0.89 | 0.94 | 2799 |
| (B) Species excluded |  |  |  |
| lm(elevation ~ temp.july + location + temp.july * location) | 0.91 | NA | 2801 |
| lm(elevation ~ temp.july + location) | 0.89 | NA | 2829 |
