# Supplementary material for: Mass elevation and lee effects markedly lift the elevational distribution of ground beetles in the Himalaya-Tibet orogen
Source: PLoS One. 2017 Mar 24;12(3):e0172939. doi: 10.1371/journal.pone.0172939 (PMC5365098; doi:10.1371/journal.pone.0172939)
Supplement: S3 Table — Comparison of random effect structures of models with radiation that either (A) included species (N = 118) or (B) excluded species (N = 232). (DOCX) [file pone.0172939.s003.docx]

**Table S3** Comparison of random effect structures for models that either (A) included species (*N* = 118) or (B) excluded species (*N* = 232)

| Model | Marginal R² | Conditional R² | AIC |
| --- | --- | --- | --- |
| (A) Species included | | | |
| lmer(elevation ~ radiation.july + (1\|Carabidae_species)) | 0.57 | 0.78 | 3058 |
| (B) Species excluded | | | |
| lm(elevation ~ temp.july) | 0.65 | NA | 3098 |
